# Endoscopic application of *n*-butyl-2-cyanoacrylate on esophagojejunal anastomotic leak: a case report

**DOI:** 10.1186/1752-1947-5-96

**Published:** 2011-03-10

**Authors:** Manousos-Georgios Pramateftakis, Georgios Vrakas, Ioannis Kanellos, Ioannis Mantzoros, Stamatis Angelopoulos, Efthymios Eleftheriades, Charalampos Lazarides

**Affiliations:** 1 4th Surgical Department, Aristotle University of Thessaloniki, Thessaloniki, Greece; 2 Aristotle University of Thessaloniki, Thessaloniki, Greece

## Abstract

**Introduction:**

This case report describes an esophagojejunal anastomotic leak following total gastrectomy for gastric cancer. The leak was treated successfully with endoscopic application of *n*-butyl-2-cyanoacrylate. This is the first case report on the endoscopic application of cyanoacrylate alone for the treatment of an anastomotic leak.

**Case presentation:**

This report describes a case of a 68-year-old Caucasian man who underwent surgery for gastric cancer. He underwent total gastrectomy and esophagojejunal anastomosis with Roux-en-Y anastomosis plus transverse colectomy. An anastomotic leak was treated conservatively at first for a total of three weeks. However, the leak persisted; therefore, the decision was made to apply topical endoscopic *n*-butyl-2-cyanoacrylate.

**Conclusion:**

The endoscopic application of *n*-butyl-2-cyanoacrylate alone can be used successfully to treat esophagojejunal anastomotic leakage.

## Introduction

Esophagojejunal anastomotic leakage is a serious complication following total gastrectomy. Studies report a frequency between 4% and 16% [1-5]. Once a leak is identified, the surgeon has to decide whether to follow conservative or surgical treatment. The conservative treatment remains drainage, parenteral nutrition and antibiotics. The endoscopic application of several tissue adhesives, such as Human Fibrin Glue can seal the anastomotic leak site. On the basis of the available bibliography, no studies to date have reported the use of *n*-butyl-2-cyanoacrylate for this purpose. The aim of our study is to present the case of an esophagojejunal anastomotic leak that was treated successfully with the topical endoscopic application of *n*-butyl-2-cyanoacrylate.

## Case presentation

**Figure 1:**
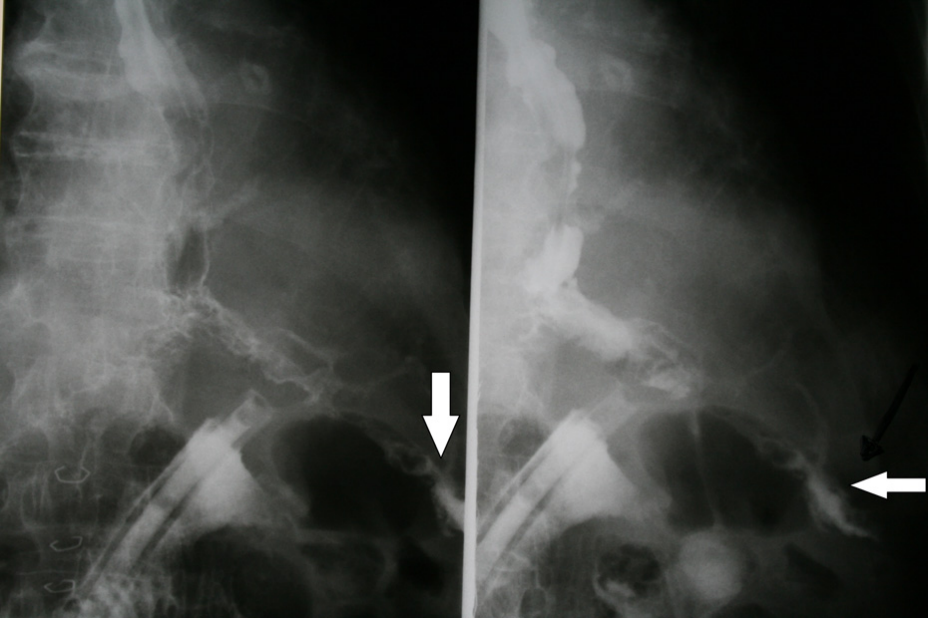
**Esophagojejunal anastomotic leak (Gastrografin swallow)**. The arrow indicates the leak.

We present the case of a 68-year-old Caucasian man who underwent surgery for gastric cancer. The tumor was arising from the pylorus and was extending higher up to the lesser curvature of the stomach. The computed tomography (CT) scan revealed infiltration of the transverse mesocolon. Therefore, the patient underwent total gastrectomy and esophagojejunal anastomosis (EEA 25 circular stapler) with Roux-en-Y anastomosis plus transverse colectomy. Both the jejunojejunal and colon anastomoses were performed in one layer with interrupted 3-0 Vicryl sutures. On the seventh postoperative day, we tested the anastomosis with radiographic studies using gastrograffin, which revealed a leak from the esophagojejunal anastomosis (Figure 1).

**Figure 2:**
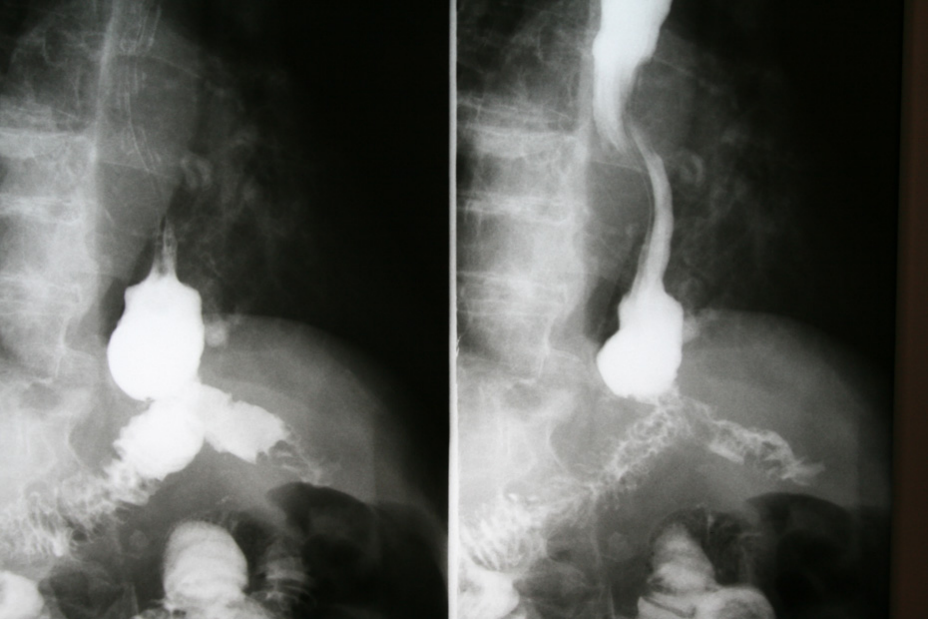
**Post-*n*-butyl-2-cyanoacrylate application**. No leak is seen.

Initially, we attempted conservative management of the leak, namely, antibiotics, food deprivation and total parenteral nutrition for a period of two weeks. The drain that was placed at the anastomotic site during the operation was kept and drained daily of 400 to 700 mL of turbid fluid (Figure 1). At the end of that two-week period, abdominal CT was performed, which did not reveal any abscesses near the leak site. However, the leak persisted; therefore, the decision was made to apply endoscopic *n*-butyl-2-cyanoacrylate (Histoacryl) on the anastomotic leak site. On the 22nd postoperative day, an endoscopy was scheduled. The leak was observed under direct vision endoscopically and measured 3-4 mm. The patient underwent a total of two sessions of *n*-butyl-2-cyanoacrylate application within 48 hours, as the first session was incomplete. Four days later we performed a new gastrograffin swallow to test the anastomosis, and there were no signs of leakage (Figure 2). The patient was discharged to home three days later.

## Discussion

An anastomotic leak is a dreaded complication after a gastrointestinal procedure. After gastrointestinal surgery, it is an important postoperative event that leads to significant morbidity and mortality. Treatment of such a leak can be troublesome. Patients with anastomotic leaks usually present with abdominal pain, tachycardia, fever, distension and leukocytosis after the fifth postoperative day. Contrast-enhanced CT and water-soluble contrast upper gastrointestinal series are diagnostic. More commonly, the leak is delayed, occurring 6-10 days postoperatively.

If the leak occurs early in the postoperative phase or the suspicion of a significant leak arises, then reoperation, peritoneal lavage and possible patching and/or resuturing may be possible. Small leaks may be managed nonoperatively if they are adequately drained. These leaks may heal spontaneously while the patient is supported with total parenteral nutrition and antibiotics [6].

One could also attempt the use of biological sealants. The important role of the biological sealants in surgery is highlighted by the long experience acquired on an international level. The literature confirms the effectiveness of biological sealants and also demonstrates the local tolerability and the absence of undesirable side effects and contraindications [7-10]. Authors often report on the favorable cost-effectiveness ratio. The latter is due to reduction of hospitalization time, rapid wound healing, early drainage removal and reduction of complications such as hematomas, sepsis, dehiscence and formation of fistulae. Because of the properties of biological sealants, they allow considerable advantages, such as the possibility of improving surgical procedures and in some cases realization of new techniques that had previously been hard to achieve [7]. The most commonly used glue for the treatment of anastomotic leaks is Human Fibrin Glue.

Biological sealants such as Human Fibrin Glue have been used to conservatively treat fistulous complications of gastrointestinal anastomoses [6-8]. In our present case, we attempted the novel use of *n*-butyl-2-cyanoacrylate on an anastomotic leak site. *n*-Butyl-2-cyanoacrylate is the first medical tissue adhesive based on cyanoacrylate. *n*-Butyl-2-cyanoacrylate is CE-marked and approved by the U.S. Food and Drug Administration. The successful application of *n*-butyl-2-cyanoacrylate has been described in other publications [9-12]. *n*-Butyl-2-cyanoacrylate's success is based upon its well-known advantages in fast wound closure and superior tensile strength. In the presence of tissue moisture, *n*-butyl-2-cyanoacrylate immediately polymerizes into a solid substance which attaches firmly to the tissue. To date, *n*-butyl-2-cyanoacrylate has been used mainly for closure of smooth and fresh skin wounds and for sclerotherapy of large esophageal or fundal varices. Furthermore, some publications have described the use of *n*-butyl-2-cyanoacrylate for gastrointestinal and vascular anastomotic leaks in rats and for recurrent congenital tracheoesophageal fistulae [9-12].

This is the first publication describing the use of *n*-butyl-2-cyanoacrylate alone on an anastomotic leak site. The treatment was successful and was followed by an excellent result. The leak healed, and the patient was allowed to eat four days following the last application of *n*-butyl-2-cyanoacrylate. Further studies are needed to test its effectiveness in comparison to more established, yet more expensive, sealants, such as Human Fibrin Glue.

## Conclusion

In conclusion, the endoscopic application of *n*-butyl-2-cyanoacrylate can successfully treat an esophagojejunal anastomotic leakage.

## Competing interests

The authors declare that they have no competing interests.

## Authors' contributions

The work presented here was carried out in collaboration among all authors. MGP and GV searched the bibliography and prepared the initial manuscript. MGP, GV and IK performed the patient's surgery. EE performed the endoscopic application of *n*-butyl-2-cyanoacrylate and contributed to writing the manuscript. IM, SA and CL contributed to the literature research and revised the initial manuscript. All authors read and approved the final manuscript.

## Consent

Written informed consent was obtained from the patient for publication of this case report and any accompanying images. A copy of the written consent is available for review by the Editor-in-Chief of this journal.
